# Intranasal Immunization of the Combined Lipooligosaccharide Conjugates Protects Mice from the Challenges with Three Serotypes of *Moraxella catarrhalis*

**DOI:** 10.1371/journal.pone.0029553

**Published:** 2011-12-22

**Authors:** Dabin Ren, Hang Xie, Wenhong Zhang, Ferdaus Hassan, Ronald S. Petralia, Shengqing Yu, David J. Lim, Xin-Xing Gu

**Affiliations:** 1 Vaccine Research Section, National Institute on Deafness and Other Communication Disorders (NIDCD), National Institutes of Health (NIH), Rockville, Maryland, United States of America; 2 Laboratory of Respiratory Viral Diseases, Office of Vaccines Research and Review, Division of Viral Products, Center for Biologics Evaluation and Research, Food and Drug Administration, Bethesda, Maryland, United States of America; 3 Section on Neurotransmitter Receptor Biology, NIDCD, NIH, Bethesda, Maryland, United States of America; 4 Section on Pathogenesis of Ear Diseases, House Ear Institute, Los Angeles, California, United States of America; Health Protection Agency, United Kingdom

## Abstract

**Background:**

There are no licensed vaccines available against *Moraxella catarrhalis*, a significant human respiratory pathogen. Lipooligosaccharide (LOS) based conjugate vaccines derived from individual serotype *M. catarrhalis* only showed partial protection coverage. A vaccine combining LOS conjugates of two or three serotypes might provide a broader protection.

**Methods:**

Mice were immunized intranasally with the combined conjugates consisting of LOS from serotype A and B or serotype A, B, and C followed by challenge with different *M. catarrhalis* strains of three serotypes. Mouse lungs, nasal washes, and sera were collected after each challenge for bacterial counts, histological evaluation, cytokine profiles, antibody level and binding activity determinations.

**Results:**

Intranasal administration of the combined LOS conjugates not only enhanced pulmonary bacterial clearance of all three serotypes of *M. catarrhalis* strains in vaccinated mice, but also elevated serotype-specific anti-LOS immunoglobulin (Ig)A and IgG titers in nasal wash and serum respectively. Mice vaccinated with the combined LOS conjugates also showed increased interferon (IFN)-γ, interleukin (IL)-12, and IL-4 in the lungs after challenges. Compared to the control group, mice immunized with the combined LOS conjugates also showed reduced lung inflammation after *M. catarrhalis* infections. The hyperimmune sera induced by the combined conjugates exhibited a broad cross-reactivity toward all three serotypes of *M. catarrhalis* under transmission electron microscopy.

**Conclusions:**

The combined vaccine of serotype A and B LOS conjugates provides protection against most *M. catarrhalis* strains by eliciting humoral and cellular immune responses.

## Introduction

*Moraxella catarrhalis* is a Gram-negative aerobic diplococcus that causes respiratory illness exclusively in humans. It is responsible for 10% – 20% of all episodes of otitis media in infants and children [1], [2]. Approximately 80% of children experience at least one episode of otitis media by the age of 3 years [3]. Otitis media accounts for 24.5 million physician visits, more than 13 million antibiotic prescriptions, and approximately $6 billion in health care costs in the United States annually [3], [4]. In addition, *M. catarrhalis* is also responsible for an estimated 2 – 4 million exacerbations of chronic obstructive pulmonary disease (COPD) in the elderly annually [2]. Prevention of *M. catarrhalis* infections by effective vaccination thus would potentially have a significant impact on both public health and the economy.

**Figure 1:**
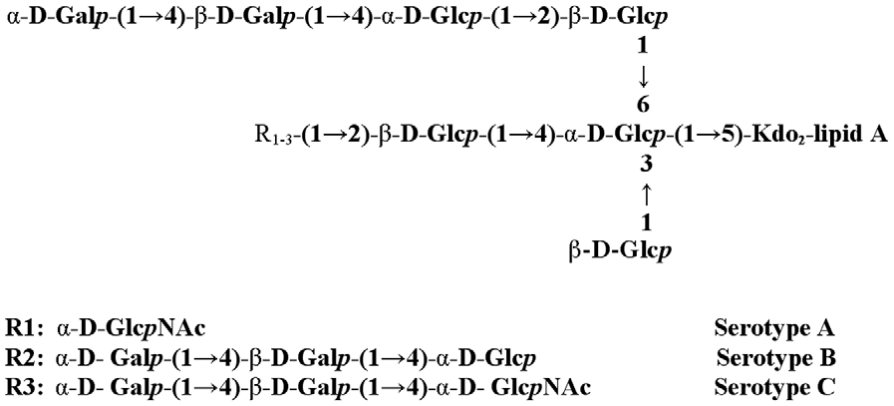
Schematic structures of the LOS moieties on the surface of *M. catarrhalis*. Three main serotypes, A, B and C, are presented with different R groups [14]–[16]. Abbreviations: Gal, galactose; Kdo, 3-deoxy-D-manno-octulosonic acid; Glc, glucose; GlcNAc, *N*-acetyl-D-glucosamine; *p*, pyranose.

However, there is no licensed vaccine for *M. catarrhalis* except that a number of *M*. *catarrhalis* vaccine candidates are under development or clinical testing [5]–[7]. Most of these vaccine candidates are designed to target adhesion molecules in the outer membrane of *M*. *catarrhalis* such as *M. catarrhalis* immunoglobulin D-binding protein (MID) [8], the ubiquitous surface protein A (UspA) [9], and catarrhalis outer membrane protein B (CopB) [10]. Although these outer membrane protein-based vaccine candidates are immunogenic, their efficiency is limited by antigenic heterogeneity [5]. The lipooligosaccharide (LOS) is the carbohydrate structure in the outer membrane of *M*. *catarrhalis*. Being a major virulence factor of *M. catarrhalis,* LOS induces excessive inflammation via a Toll-like receptor 4 (TLR4) and CD14 dependent pathway [11]. The structures of LOS are conserved among 95% of known *M. catarrhalis* strains and clinical isolates [12], [13]. Based on the LOS structures (Figure 1) [14]–[16], *M. catarrhalis* can be categorized into three serotypes: A, B, and C accounting for 61.3%, 28.8%, and 5.3% of the 302 strains tested [12]. Monoclonal antibodies specific for serotype A LOS have been reported to cross-react with serotype C LOS [13]. We have shown that *M. catarrhalis* LOS-based conjugate vaccine candidates from three individual serotypes were immunogenic in vivo, but were only able to elicit bactericidal activity toward a portion of *M. catarrhalis* strains and clinical isolates [17]–[19]. Immunization with a LOS conjugate derived from serotype A protects against homologous and heterologous challenges including serotype A strains and a serotype C strain but not a serotype B strain in a mouse pulmonary clearance model [20], [21]. Similarly, immunization with a LOS conjugate derived from serotype B or C alone has been shown to protect against only partial *M. catarrhalis* strains in our preliminary mouse pulmonary clearance study.

Based on these findings, we hypothesized that a combination of LOS conjugates from two or three serotypes would protect against most *M. catarrhalis* strains. To test this, we vaccinated mice with the combined *M. catarrhalis* LOS conjugates consisting of serotype A and B or serotype A, B, and C via intranasal route. The protection elicited by the combined LOS conjugates against homologous and heterologous strains of *M. catarrhalis* was evaluated in a mouse pulmonary clearance model. Our primary goal was to determine the optimal conjugate combination with the maximum protection against all three serotypes of *M. catarrhalis* in mice.

## Materials and Methods

### Ethics statement

All experiments involving mice were performed according to the recommendations in the Guide for the Care and Use of Laboratory Animals of the National Institutes of Health. Protocols were reviewed and approved by institutional review boards at the National Institutes of Health (Permit Number: 1158).

### Bacterial strains

*M. catarrhalis* strain O35E (serotype A) was kindly provided by Eric J. Hansen (the University of Texas, Dallas, TX) and strain 25238 (serotype A) was purchased from the American Type Culture Collection (Manassas, VA). *M. catarrhalis* serotype B strains 26397 and 26400, and serotype C strains 26404 and 26391 were obtained from the Culture Collection of the University of Goteborg, Department of Clinical Bacteriology, Goteborg, Sweden.

### Conjugate vaccines

Purification of LOSs from prototype strains 25238 (A), 26397 (B), and 26404 (C), detoxification of the LOSs, and conjugation of detoxified LOSs (dLOSs) to the carrier tetanus toxoid (TT) were performed for each serotype LOS individually as described [17]–[19]. The synthesized dLOS-TT from 25238, 26397, or 26404 was designated as conjugate A, B, or C, respectively. The composition of the conjugates is described in Table 1. The combination of conjugate A physically mixed with conjugate B in the equal amount and the combination of conjugate A physically mixed with conjugate B and conjugate C in the equal amount were named as conjugates AB and ABC, respectively.

**Table 1:** Composition of conjugate vaccines.

Conjugate	Concentration (µg/ml)		dLOS/TT molar ratio^a^
	dLOS	TT	
25238 dLOS-TT (A)	318.9	419.4	38
26397 dLOS-TT (B)	665.2	700.6	47
26404 dLOS-TT (C)	503.3	659.6	38

a Expressed as moles of dLOS per mole of TT protein, with molecular weights of 3,000 Da for dLOS and 150,000 Da for TT.

### Immunizations

Six to 8 weeks old female BALB/c mice were obtained from Taconic Farms Inc. (Germantown, NY). Cholera toxin (List Biological Laboratories, Campbell, CA) was used as the mucosal adjuvant at 1 µg/dose/mouse either alone or in the combination with conjugates AB, or conjugates ABC (2.5 µg of each carbohydrate per dose per mouse). Mice (8 – 10 per group) were anesthetized intraperitoneally with 0.1 ml of 2% ketamine (Fort Dodge Laboratories, Inc., Fort Dodge, IA) and 0.2% xylazine (Miles Inc., Shawnee Mission, KS) in 0.9% NaCl. A total of 18 µl of each vaccine preparation was slowly instilled through a micropipette into both nares of each mouse. Mice were immunized weekly for a total of 4 weeks.

### Bacterial challenge and sampling

One week after the last booster, mice were challenged with aerosolized *M. catarrhalis* strains 25238 (A), O35E (A), 26397 (B), 26400 (B), 26404 (C), or 26391 (C) at 5×10^8^ to 1×10^9^ CFU/ml in an inhalation exposure system (Glas-Col, LLC, Terre Haute, IN) as described [22]. At 6 h post-challenge, mice were euthanized. Sera and nasal washes were collected for antibody detection. A subset of mouse lungs were harvested and homogenized in 1 ml of phosphate buffered saline (PBS) with a Precellys 24 homogenizer (Bertin Technologies, Paris, France). Serially diluted lung homogenates were cultured on chocolate agar plates overnight at 37°C with 5% CO_2_ to determine colony forming unit (CFU) counts. The rest of lung homogenates were then frozen for cytokine profiles. Another subset of mouse lungs were harvested and fixed in 10% neutral formaldehyde for histological assessment.

### Enzyme-linked immunosorbent assay (ELISA)

The 96-well plates were coated with LOS of strains 25238 (A), 26397 (B), or 26404 (C) at 10 µg/ml. The anti-LOS specific IgA in nasal washes and IgG in sera were determined by ELISA as described [17] and were expressed as the reciprocal of the highest serum dilution. Specific mouse hyperimmune sera against the whole cells of strain 25238 (A), 26397 (B), or 26404 (C) were used as references [17]–[19].

### Cytokine determination

The inflammatory cytokines including interferon (IFN)-γ, interleukin (IL)-12 (total), IL-4, tumor necrosis factor (TNF)-α, and mouse keratinocyte chemoattractant (mKC) in the lung homogenates following challenges were quantified using a mouse TH1/TH2 9-Plex Assay Ultra-Sensitive Kit according to the manufacturer's instructions and analyzed in a SECTOR imager 2400 (Meso Scale Discovery, Gaithersburg, MD).

### Histological evaluation

Fixed mouse lungs were embedded in paraffin, sectioned, mounted on slides, and stained with hematoxylin and eosin (H&E). The slides were then observed and imaged under an Olympus IX71 microscope with a digital camera (Olympus America Inc., Miami, FL). Lung sections were scored for lung injury, including the following: (1) intra-alveolar infiltration of inflammatory cells, (2) thickness of the alveolar wall, (3) alveolar hemorrhage, and (4) intra-alveolar proteinaceous exudate. The items were semi-quantitatively scored as none, minimal, light, moderate, or severe (score 0, 1, 2, 3, or 4, respectively) by a pathologist blinded to the experimental groups. The combined score of all four parameters was taken for each field. Ten randomly chosen fields from each animal with approximately the same number of alveoli were analyzed on each slide at ×400 magnification. The lung injury score was obtained by averaging the scores of all the fields from each animal and 6 mice per group were inspected.

### Immunoelectron microscopy of bacteria

Formvar-coated 200-mesh grids (Electron Microscopy Sciences, Hatfield, PA) were floated on the suspension of strain 25238 (A), 26397 (B), or 26404 (C) in PBS and blotted dry. Grids were then incubated with sera from mice intranasally immunized with the combined conjugates AB or the corresponding pre-immune sera (1∶400 dilution in PBS with 0.5% BSA) at room temperature for 30 min. Subsequently, the grids were incubated with gold (5-nm diameter)-conjugated goat anti-mouse IgG (Ted Pella, Inc., Redding, CA, 1∶20 dilution in PBS with 0.5% BSA) for 60 min. The grids were blotted and washed 3 times with PBS between steps. The bacteria were then negatively stained with a mixture of equal volumes of 2% ammonium acetate and 2% ammonium molybdate (Sigma, St. Louis, MO). The grids were viewed under a JEOL JEM-1010 transmission electron microscope (JEOL Ltd., Tokyo, Japan).

### Statistical analysis

The geometric mean (GM) of the anti-LOS specific IgA and IgG titers from n independent observations plus/minus (±) standard deviations (SD) were determined. The pulmonary levels of viable residual bacteria counts were logarithm transformed ((log)_10_ CFU) before being subjected to one-way analysis of variance (ANOVA). The differences in the lung cytokines and the differences in the pathological lung injury scores among different groups were determined by ANOVA. A *P* value less than 0.05 was considered significant. A nonparametric Spearman correlation test was employed to analyze the correlations among the residual bacterial counts, antibodies, and pulmonary cytokine levels.

## Results

### Bacterial clearance in the mouse lungs

**Figure 2:**
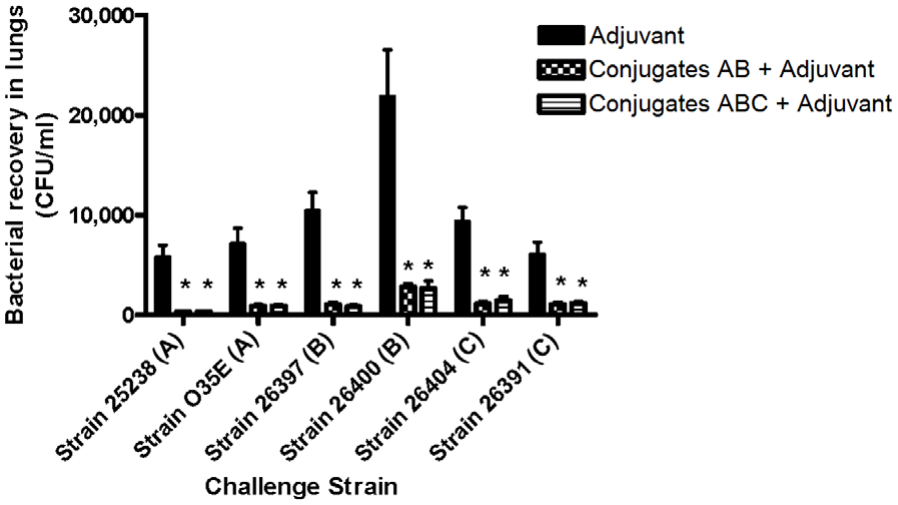
Pulmonary bacterial recovery after challenges with three serotypes of *M. catarrhalis*. Mice (8 – 10 per group) were vaccinated intranasally (1 dose per week, 4 doses in total) with either the adjuvant cholera toxin alone (1 µg/dose/mouse) or the combined conjugates AB or ABC (2.5 µg of each carbohydrate per dose per mouse) plus adjuvant. One week after the last booster, mice were challenged with aerosolized *M. catarrhalis* including 25238 (type A), O35E (type A), 26397 (type B), 26400 (type B), 26404 (type C), or 26391 (type C) (5×10^8^ to 1×10^9^ CFU/ml). Mouse lungs were harvested at 6 h after the challenge for bacterial counts. The viable bacteria are expressed as the mean of CFUs ± standard error of the mean (SEM). *P<0.05 indicates the significant difference vs adjuvant control by ANOVA.

Figure 2 shows the pulmonary bacterial clearance in immunized mice after challenge with different *M. catarrhalis* strains (Fig. 2). Compared to adjuvant immunized control group, intranasal vaccination with the combined conjugates AB or ABC plus adjuvant significantly reduced the pulmonary bacterial burdens by 84% – 96% in immunized mice following challenges with three serotypes of *M. catarrhalis* (Fig. 2). However, the addition of conjugate C in the vaccine mixture showed no obvious benefit in pulmonary clearance of *M. catarrhalis* including serotype C strains when compared to the mice receiving only the combined conjugates AB (Fig. 2).

### Mucosal and systemic antibody responses

Intranasal immunization of the combined LOS conjugates plus adjuvant significantly increased anti-*M. catarrhalis* LOS serotype-specific IgA levels in nasal wash (conjugates AB plus adjuvant vs adjuvant alone: 2.8 – 7.7 folds; conjugates ABC plus adjuvant vs adjuvant alone: 5.4 – 28.4 folds) following challenges (Table 2). Furthermore, intranasal administration of the combined LOS conjugates plus adjuvant also enhanced serotype-specific IgG levels in serum (conjugates AB plus adjuvant vs adjuvant alone: 2.9 – 10.0 folds; conjugates ABC plus adjuvant vs adjuvant alone: 8.6 – 34.1 folds). The higher levels of nasal IgA and serum IgG produced, the lower levels of viable bacteria recovered in the lungs of the vaccinated mice following challenges with all three serotypes of *M. catarrhalis* strains (r = −0.3532, *P*<0.0001 for nasal IgA; r = −0.4195, *P*<0.0001 for serum IgG). In general, administration of the combined conjugates ABC elicited higher levels of anti-*M. catarrhalis* LOS specific IgA in nasal wash and IgG in serum than the combined conjugates AB (Table 2).

**Table 2:** Antibody responses of mice vaccinated with the combined conjugates after challenges with *M. catarrhalis* strains.

Challenge Strain	Antibody class	GM (± SD range) of antibody titers^a^		
		Adjuvant	Conjugates AB+Adjuvant	Conjugates ABC+Adjuvant
25238 (A)	Nasal wash IgA	1.3 (0.8–2.2)	8.1 (0.4–183.8)	7.2 (1.4–36.6)
	Serum IgG	1.0 (1.0)	6.5 (0.4–116.0)	33.6 (1.7–653.0)
O35E (A)	Nasal wash IgA	1.0 (1.0)	3.6 (0.9–14.5)	28.4 (2.3–343.7)
	Serum IgG	1.3 (0.6–2.9)	4.3 (0.4–50.5)	41.9 (2.6–688.0)
26397 (B)	Nasal wash IgA	1.5 (0.9–2.7)	4.2 (1.1–16.5)	8.1 (0.9–70.2)
	Serum IgG	1.1 (0.8–1.7)	7.2 (0.4–134.0)	17.4 (0.3–904.4)
26400 (B)	Nasal wash IgA	1.1 (0.8–1.7)	3.3 (0.4–25.6)	15.6 (1.1–221.4)
	Serum IgG	1.0 (1.0)	10.0 (0.5–200.8)	19.4 (0.4–874.6)
26404 (C)	Nasal wash IgA	2.6 (0.6–11.6)	14.7 (1.7–124.1)	65.0 (7.3–578.1)
	Serum IgG	1.1 (0.8–1.7)	4.3 (0.3–67.4)	37.5 (3.7–381.6)
26391 (C)	Nasal wash IgA	1.5 (0.7–3.4)	11.5 (1.2–109.0)	30.1 (2.0–454.4)
	Serum IgG	1.3 (0.8–2.2)	3.8 (0.6–25.2)	11.2 (1.5–82.2)

a Mice (8 – 10 per group) were vaccinated intranasally (1 dose per week, 4 doses in total) with either the adjuvant cholera toxin alone (1 µg/dose/mouse) or the combined conjugates AB or ABC (2.5 µg of each carbohydrate per dose per mouse) plus adjuvant. One week after the last vaccination, mice were challenged with an aerosol of indicated *M. catarrhalis* strain (5×10^8^ to 1×10^9^ CFU/ml). The nasal washes and sera were harvested at 6 h after the challenge for antibody detection. Data are presented as geometric mean (GM) titer ± standard deviation (SD).

### Pulmonary cytokine secretion after challenges

**Figure 3:**
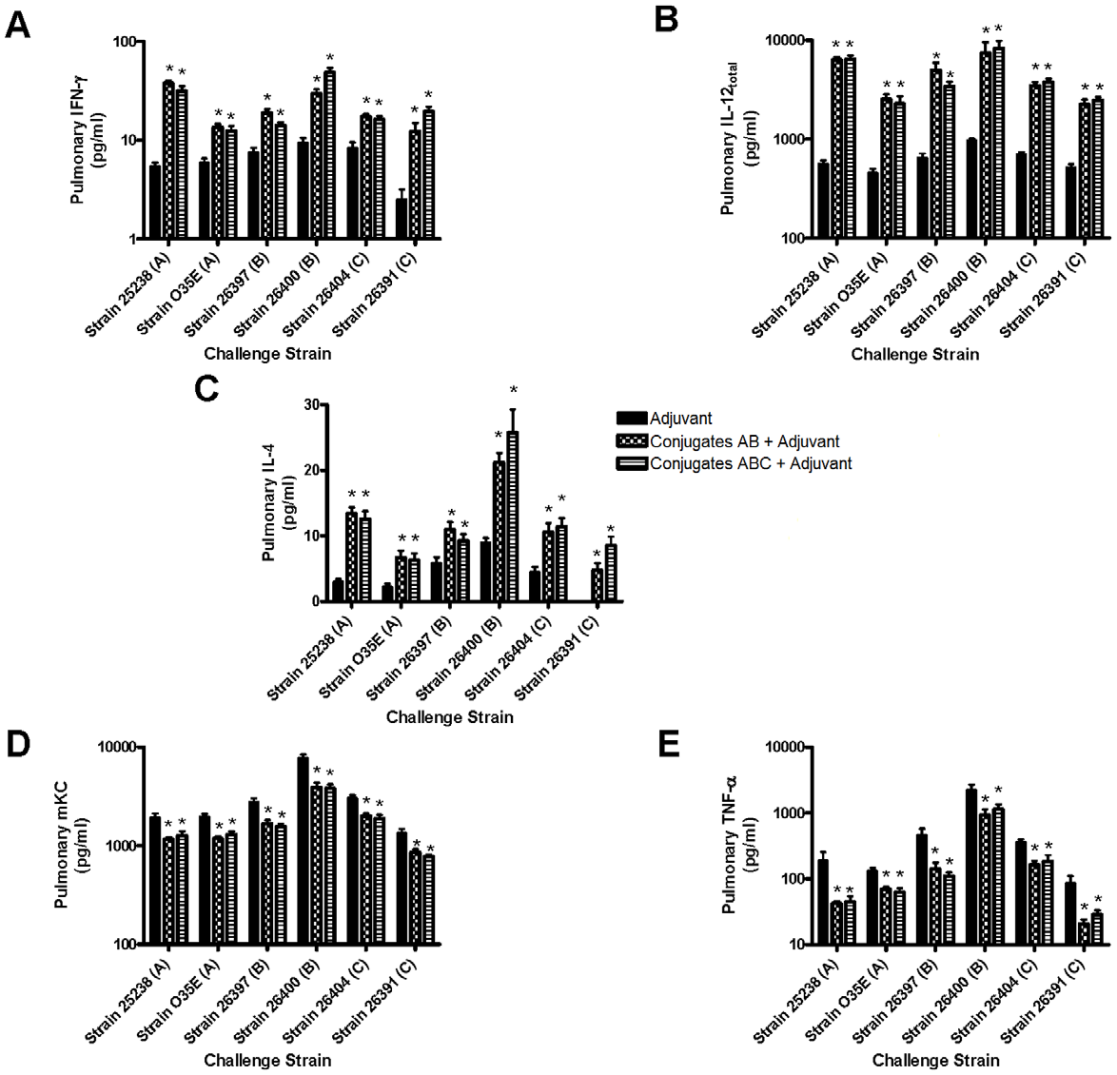
Pulmonary cytokine levels after challenges with three serotypes of *M. catarrhalis*. Mice were vaccinated (1 dose per week, 4 doses in total) with either the adjuvant cholera toxin alone (1 µg/dose/mouse) or the combined conjugates AB or ABC (2.5 µg of each carbohydrate per dose per mouse) plus adjuvant. One week after the last booster, mice were challenged with aerosolized *M. catarrhalis* including 25238 (type A), O35E (type A), 26397 (type B), 26400 (type B), 26404 (type C), or 26391 (type C) (5×10^8^ to 1×10^9^ CFU/ml). At 6 h after the challenge, mouse lungs were harvested and homogenized for detection of IFN-γ, IL-12, IL-4, mKC, and TNF-α. Data are presented as mean ± SEM. *P<0.05 indicates the significant difference vs adjuvant control by ANOVA. Note: Pulmonary IL-4 in mice immunized with adjuvant only was below the detection limit after *M. catarrhalis* 26391 challenge.

Following challenges with three serotypes of *M. catarrahlis* strains, the pulmonary levels of IFN-γ, IL-12, and IL-4 were dramatically elevated in the mice receiving the intranasal vaccination of the combined LOS conjugates plus adjuvant (Fig. 3A, 3B, and 3C). Meanwhile, the levels of mKC and TNF-α in the same lung homogenates were also significantly reduced when compared to adjuvant administered controls (Fig. 3D and 3E). The elevated pulmonary IFN-γ, IL-12, and IL-4 levels positively correlated with the increased nasal wash IgA titers (r = 0.2152, *P*<0.01 for IFN-γ; r = 0.3060, *P*<0.001 for IL-12) and serum IgG titers (r = 0.3034, *P*<0. 001 for IFN-γ; r = 0.3707, *P*<0.0001 for IL-12; r = 0.2032, *P*<0.05 for IL-4) in mice that had been immunized with the combined conjugates AB or ABC plus adjuvant. Moreover, the reduced pulmonary levels of inflammatory mKC and TNF-α also significantly correlated with the decreased bacterial recovery in the same lung homogenates of the mice receiving the combined LOS conjugates plus adjuvant (r = 0.6380, *P*<0.0001 for mKC; r = 0.6239, *P*<0.0001 for TNF-α).

### Histological changes in the lungs of vaccinated mice after challenges

**Figure 4:**
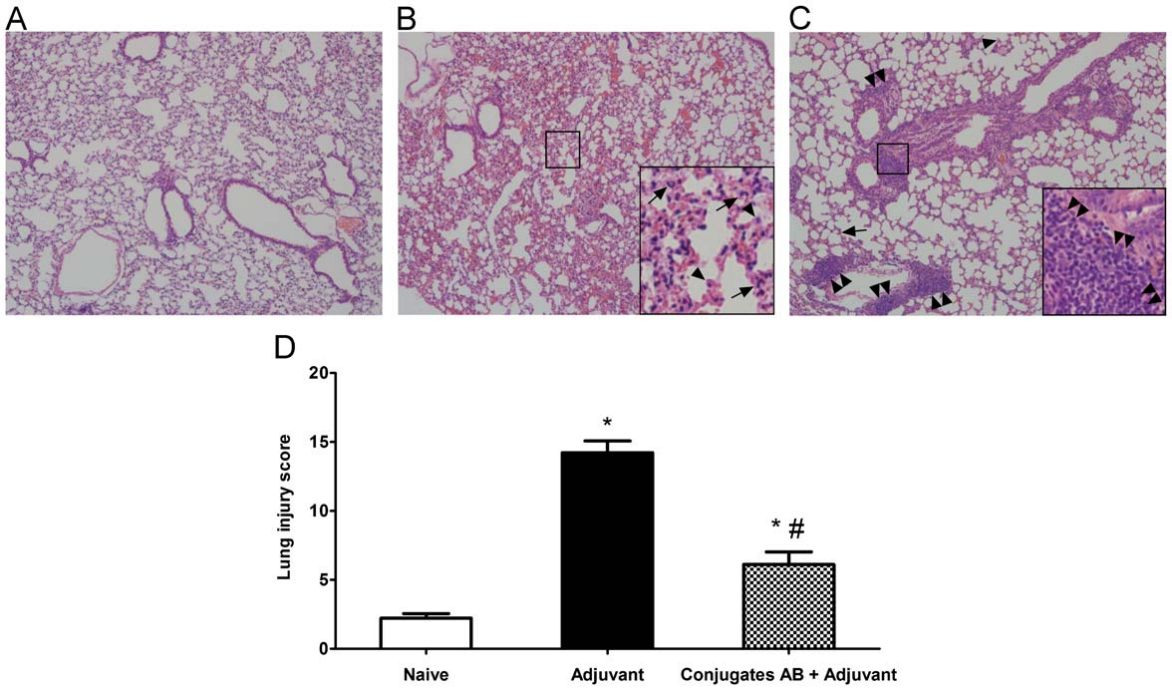
Histopathology of mouse lungs after challenge with *M. catarrhalis*. Mice were vaccinated intranasally (1 dose per week, 4 doses in total) with either the adjuvant cholera toxin alone (1 µg/dose/mouse) or the combined conjugates AB (2.5 µg of each carbohydrate per dose per mouse) plus adjuvant. One week after the last booster, mice were challenged with aerosolized *M. catarrhalis*. Mouse lungs were harvested 6 h later for H&E staining. (A) Normal lung of a naive mouse; (B) Lung of the mouse administered with adjuvant only; (C) Lung of the mouse vaccinated with the combined conjugates AB plus adjuvant. **Arrows** and **arrowheads** show infiltrating neutrophils and macrophages, respectively, in inflamed lungs of mice after challenge by *M. catarrhalis* strains. **Double arrowheads** show proliferating lymphocytes in the lungs of conjugate-immunized mice after challenge by *M. catarrhalis* strains. Original magnification ×100; ×400 (**insets**). (D) Histopathological lung injury scores for mice receiving either the adjuvant only or the combined conjugates AB plus adjuvant followed with *M. catarrhalis* challenges. The scores were obtained from 10 random fields per mouse and 6 mice per group at magnification ×400. The categories used to generate the score were intra-alveolar infiltrates, alveolar septal thickeness, alveolar hemorrhage, and intra-alveolar proteinaceous exudates. Data are expressed as mean ± SD. *P<0.05 or ^#^ *P*<0.05 indicates the significant difference vs naive mice or adjuvant only administered mice by ANOVA, respectively.

Unlike the normal mouse lung architecture (Fig. 4A), the lungs of the adjuvant alone administered mice exhibited prominent infiltration of neutrophils and macrophages after *M. catarrhalis* challenges (Fig. 4B). However, the *M. catarrhalis* induced pulmonary infiltration of inflammatory cells was diminished in the mice that had been vaccinated with the combined conjugates AB plus adjuvant (Fig. 4C). Consistently, the adjuvant alone immunized mice displayed severe lung injury when compared to naive mice (*P*<0.05) (Fig. 4D). On the contrary, vaccination with the combined conjugates AB plus adjuvant significantly reduced the score of acute lung injury in mice (*P*<0.05 vs adjuvant alone, Fig. 4D) following *M. catarrhalis* challenges. In addition, a robust lymphoproliferation along with the formation of focally propagated lymphocytes were observed in the subepithelia of trachea and bronchi of the mice immunized with the conjugates AB plus adjuvant followed by *M. catarrhalis* challenges (Fig. 4C). In contrast, the lymphocytic proliferation was not found in the lungs of naive mice and the lungs of adjuvant only immunized mice after the challenges (Fig. 4A and 4B). The mice vaccinated with the conjugates ABC plus adjuvant showed the similar lung histological changes as those of the mice receiving the conjugates AB plus adjuvant after *M. catarrhalis* challenges (data not shown).

### Immunoelectron microscopy of *M. catarrhalis*

**Figure 5:**
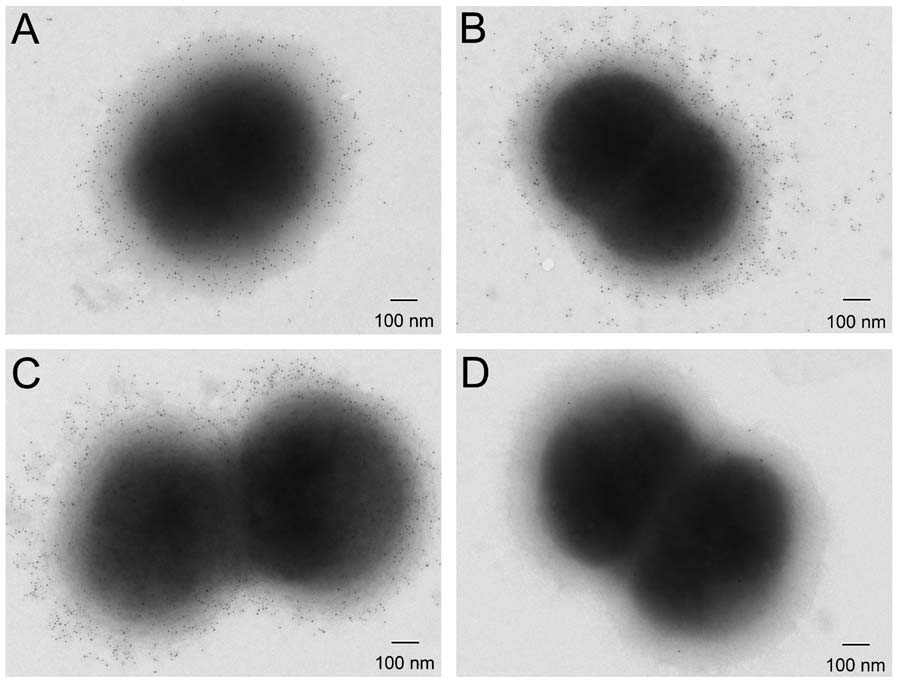
Immuno-electron microscopy of *M. catarrhalis*. Mice were vaccinated intranasally (1 dose per week, 4 doses in total) with either the adjuvant cholera toxin alone (1 μg/dose/mouse) or the combined conjugates AB (2.5 µg of each carbohydrate per dose per mouse) plus adjuvant. Mouse sera were collected at 1 week after the final immunization. Whole cells of strain 25238 (type A, A), 26397 (type B, B), or 26404 (type C, C) were incubated with the mouse hyperimmune sera (1:400 dilution) followed by gold (5-nm diameter)-conjugated goat anti-mouse IgG. Whole cells of strain 25238 (D) incubated with the corresponding pre-immune mouse sera (1:400 dilution) were included as a control. Scale bar, 100 nm.

The cross-reactivity of anti-*M. catarrhalis* LOS specific hyperimmune mouse sera generated by vaccination with the combined conjugates AB plus adjuvant were further characterized by immunoelectron microscopy. As shown in Fig. 5, electron-dense gold particles clearly scattered across the surfaces of all three serotypes of *M. catarrhalis* strains including 25238 (type A, Fig. 5A), 26397 (type B, Fig. 5B), and 26404 (type C, Fig. 5C) after pre-incubation with the hyperimmune sera of mice vaccinated with the combined conjugates AB plus adjuvant. In contrast, the whole cells of 25238 pre-incubated with the corresponding mouse pre-immune sera showed no binding of gold particles on the surface (Fig. 5D).

## Discussion

LOS is a major virulence factor located in the outer membrane of *M. catarrhalis*. It can potentially induce excessive inflammation via cellular cross-talk [11]. Unlike the outer membrane proteins such as MID, UspA, and CopB that show a great plasticity in their antigenicity [6], [23], the structures of LOS are highly conserved within each of the three serotypes of *M. catarrhalis* [12], [13]. Thus LOS has the great potential to be developed into carbohydrate-based conjugate vaccines against *M. catarrhalis* infections. Previously we have reported that LOS based conjugate vaccines derived from individual serotypes of *M. catarrhalis* were highly immunogenic in vivo, but could protect against only a fraction of *M. catarrhalis* strains [17]–[21]. Hence, we hypothesized that a combination of LOS conjugates of two or three serotypes could provide a broader protection against most of the known *M. catarrhalis* strains.

In the present study, we demonstrated that intranasal immunization of the combined LOS conjugates from serotype A and B or from serotype A, B, and C led to a significant reduction in the bacterial burden in the lungs of *M. catarrhalis* infected mice. Mice vaccinated with the combined LOS conjugates not only efficiently cleared *M. catarrhalis* of the same serotype out of the respiratory system but also the strains of different serotypes. Interestingly, both the combined LOS conjugates AB and ABC demonstrated a similar efficiency in clearing not only serotype A and B strains but also serotype C bacteria. This is not surprising since there is a high cross-reactivity in the antibodies against the LOS structures between serotype A and C strains [13]. Serotype A and B *M. catarrhalis* are the major clinical isolates and serotype C comprises less than 6% of *M. catarrhalis* strains identified so far. The supplement of serotype C conjugate into conjugates AB combination may induce additional homologous or cross-reactive heterologous antibodies against each serotype LOS antigen. Therefore, the mice receiving the ABC conjugates generally showed higher levels of serum IgG and nasal IgA responses than the mice administered with the AB conjugates. However, our results indicated that intranasal vaccination with the combined conjugates consisting of serotype A and B LOS was efficient to combat the tested *M. catarrhalis* strains of all three serotypes. The addition of serotype C conjugate into the conjugates AB combination did not further enhance the efficiency of pulmonary bacterial clearance in the current study. Our results also suggest that the combined LOS conjugates AB could be a potent and cost-effective mucosal vaccine candidate to fight against *M. catarrhalis* infections.

As revealed by the histology data, intranasal immunization of the combined LOS conjugates also resulted in a robust proliferation of mucosal lymphocytes in the airway branches in vaccinated mice. These mucosal lymphocytes could potentially differentiate into polymeric IgA (pIgA)-producing plasma cells and produce secretory IgA (SIgA) [24], [25]. Anti-antigen specific SIgA could enhance adherence of bacteria to mucus, thereby promoting clearance by respiratory ciliary movement [24]. SIgA can also inhibit bacterial agglutination, adherence for epithelial colonization and penetration for invasion [26]. In addition to elevated mucosal IgA secretion, intranasal immunization of the combined LOS conjugates also significantly increased anti-antigen specific serum IgG. The induced anti-LOS specific serum IgG demonstrated a broad cross-reactivity toward all three prototypes of *M. catarrhalis* strains under electron microscopy. Highly cross-reactive serum IgG might extravasate into the infected respiratory mucosal surface, promote phagocytosis of *M. catarrhalis* by alveolar macrophages and infiltrating neutrophils, and facilitate bacterial clearance by complement-mediated killing [27].

In addition to humoral responses, intranasal immunization of the combined LOS conjugates also stimulated anti-antigen specific cell-mediated immune responses in the lungs of vaccinated mice. Pulmonary augmentation of Th1 type cytokines IFN-γ and IL-12 could promote phagocytosis and respiratory burst thus leading to killing of *M. catarrhalis* [28]. IFN-γ could also induce the antibody isotype µ→γ2α switch and result in IgG2a antibody production that could potentially enhance bacterial clearance by complement-mediated cytotoxicity [29], [30]. Similarly, pulmonary IL-4, a typical Th2 type cytokine was also significantly increased in the combined conjugates vaccinated mice following challenges. IL-4 is pivotal in B-cell switching from secretory IgM (SIgM) to IgG_1_ subclass production and promotes IgG_1_, IgG2b, and IgA responses in mice [29]. The augmentation of IL-4 has been linked to enhanced *M. catarrhalis* clearance from mouse lungs [28]. In our present study, enhanced pulmonary Th1 and Th2 cytokines not only correlated significantly with increased lung clearance of *M. catarrhalis*, but also correlated well with elevated mucosal IgA and serum IgG titers.

Moreover, intranasal immunization of the combined LOS conjugates plus adjuvant also resulted in significantly less pro-inflammatory cytokines such as TNF-α and mKC in the lungs of vaccinated mice after *M. catarrhalis* infections than those administered with adjuvant only. The reduced pro-inflammatory TNF-α and mKC correlated significantly with enhanced pulmonary clearance of *M. catarrhalis*, suggesting vaccination of the combined LOS conjugates could reduce *M. catarrhalis* induced pulmonary inflammation. This was consistent with the histology data showing that the combined conjugates vaccinated mouse lung structures were more like those of the normal mouse lung.

In summary, intranasal vaccination with the combined conjugates consisting of LOS from serotype A and B could efficiently protect mice from the challenges of tested *M. catarrhalis* strains of all three serotypes.
